# The ideal-reality gap: a qualitative study of nurse middle managers' perspectives on speaking up for patient safety

**DOI:** 10.3389/fpubh.2026.1790424

**Published:** 2026-04-09

**Authors:** Jiahui Dong, Li Du, Jiangtao Xu, Jing Li, Jingze Tao, Fang Wang

**Affiliations:** 1 School of Nursing, Nanjing University of Chinese Medicine, Nanjing, Jiangsu, China; 2 School of Nursing, Bengbu Medical University, Bengbu, Anhui, China; 3 Qinhuai Medical District, Jinling Clinical Medical College, Nanjing University of Chinese Medicine, Nanjing, Jiangsu, China

**Keywords:** assertiveness, leadership, middle managers, nurses, patient safety, qualitative study

## Abstract

**Background:**

As pivotal figures in patient safety management, nurse managers are well-positioned to gain comprehensive insights into the barriers and concerns surrounding nurses' speaking up for patient safety (SUPS) due to their close engagement with frontline nurses. However, few studies have investigated nurse managers' perspectives on SUPS. Therefore, this study aimed to explore nurse middle managers' perceptions and recommendations regarding nurses' SUPS, to inform strategies for promoting SUPS practice.

**Methods:**

Fourteen nurse middle managers were recruited via purposive and snowball sampling from a tertiary Grade A hospital in Jiangsu Province between June and August 2025. Face-to-face interviews were conducted using a semi-structured interview guide. Data analysis was guided by reflexive thematic analysis.

**Results:**

Three themes were identified: (1) conceptualizing SUPS: Intrinsic Duty and Governance Resource; (2) the Ideal-Reality gap: the Dilemma of SUPS Under Multiple Constraints; and (3) The Key to Empowerment: Building a Supportive Ecosystem. Ten subthemes underscored these findings.

**Conclusion:**

SUPS serves as a professional duty and a critical governance resource. However, a pronounced “ideal-reality gap” persists, driven by multilevel factors rather than mere individual hesitancy. Consequently, promoting SUPS requires not only training in individual assertiveness but also engineering a resilient institutional ecosystem that incorporates non-punitive governance, manager empowerment, and systemic safeguards to create a supportive organizational environment that promotes speaking up.

## 1 Introduction

Patient safety constitutes a fundamental tenet of hospital quality management, aimed at minimizing healthcare-associated harm to an acceptable level. Despite its prioritization, the safety landscape presents a persistent public health challenge. A recent WHO publication (1) indicated that approximately 134 million adverse events occur annually due to unsafe care, precipitating nearly 3 million deaths and substantial economic burdens, with over half of these harms being preventable. In addressing this crisis, transparent communication is recognized as a linchpin for organizational improvement and health system resilience (1, 2). Within this framework, Speaking Up for Patient Safety (SUPS), defined as assertive and change-oriented communication in which professionals proactively articulate safety concerns or improvement suggestions to relevant stakeholders upon identifying hazards, has emerged as a critical safety mechanism (3). Highlighting this imperative, the WHO adopted “Speak up for patient safety” as the slogan for the inaugural World Patient Safety Day in 2019 (4). Beyond being a subset of “employee voice,” SUPS functions as a vital organizational citizenship behavior with a distinct safety orientation (5, 6), serving as a vital predictor of patient safety outcomes and a barrier against systemic failure (7, 8).

Positioned at the clinical forefront, nurses constitute the primary defense in identifying and intercepting safety risks, often functioning as the “eyes and ears” of healthcare institutions (5). Effective speaking up facilitates real-time information exchange and hazard intervention, thereby mitigating adverse events such as medication errors and hospital-acquired infections, optimizing care systems, and yielding positive health economic outcomes (9, 10). However, translating this mandate into practice is fraught with sociopsychological barriers. Inhibited by the fear of being labeled “troublemakers” or disrupting team cohesion, nurses frequently resort to organizational silence, inadvertently compromising patient interests (11, 12). Moreover, rigid medical hierarchies and cultural norms prioritizing harmony and conflict avoidance, particularly in East Asian Confucian contexts, further inhibit the willingness to openly articulate safety concerns (13, 14). Consequently, the decision to speak up is a situational process influenced by a complex interplay of multiple factors.

In this situational dynamic, nurse managers occupy a strategic node in hospital safety governance. Beyond their operational responsibilities, they act as the primary architects of the unit-level safety climate, directly influencing subordinates' safety motivation (15). Inclusive and empowering leadership behaviors can enhance nurses' organizational identification and willingness to speak up (16, 17), whereas autocratic or abusive managers who perceive safety inquiries as challenges to authority often foster a culture of silence (18). Empirical evidence indicates a significant positive correlation between unit communication openness, leadership support for patient safety, and nurses' voice behaviors (19). Furthermore, safety leadership styles are proven to influence subordinates' safety participation and proactive behaviors (15, 20). Thus, as the primary providers of administrative and emotional support, managers' perceptions and attitudes influence whether frontline safety concerns effectively heard and translated into actionable improvements.

Nurse middle managers occupy a unique position of “obeying upwards and managing downwards,” which affords them a holistic vantage point on the systemic facilitators and constraints surrounding SUPS. In recent years, qualitative studies in countries such as Iran and South Korea have explored this issue from the perspective of managers or senior nurses, highlighting the importance of strategies like safety rounds and non-punitive feedback in creating a psychologically safe environment (21, 22). However, in the context of traditional Chinese culture, where the Confucian philosophy of the “Golden Mean” encourages handling conflicts gently, nurses may be less inclined to directly voice safety doubts. Existing literature on SUPS among nurses in China is predominantly quantitative (23, 24) and focuses on frontline nurses, lacking in-depth qualitative exploration of the manager's perspective regarding the understanding, practical dilemmas, and management of SUPS. To address these gaps, this study is grounded in the Chinese healthcare context and focuses on nurse middle managers as key informants to enhance the understanding of SUPS among nurses. It specifically examines factors influencing SUPS among nurses and management recommendations from the managerial viewpoint. The findings will provide an evidence base for hospital administration to refine institutional support and foster a positive safety culture, thereby enriching the global theoretical understanding of safety voice management. For the sake of clarity and brevity, we refer to nurse middle managers as nurse managers in the following text.

## 2 Research objectives

The aim of the study was to analyze the perceptions and experiences of nurse managers in Chinese hospitals regarding nurses' SUPS, identify the challenges they recognize in this process, and explore potential management strategies.

## 3 Materials and methods

### 3.1 Study design

A descriptive qualitative design was employed to explore nurse managers' perceptions and managerial recommendations regarding nurses' SUPS. This methodological approach is particularly effective for capturing the factual nuances and subjective meanings embedded in participants' narratives within natural settings (25). The reporting of this study adhered to the Consolidated Criteria for Reporting Qualitative Research (COREQ) checklist (Supplementary data S1) (26).

### 3.2 Study setting and recruitment

Participants were recruited from a tertiary Grade A hospital in Jiangsu Province, China. A purposive sampling approach, supplemented by snowball sampling, was adopted to diversify perspectives and improve recruitment efficiency. Interviews were conducted in quiet, spacious, and well-lit offices or study rooms to ensure privacy and to prevent interruptions throughout the interview process. The research team comprised five master's nursing students and one expert in patient safety management, and all members had systematically completed qualitative research coursework. Following in-depth discussions and considering the distinctive nursing leadership structure in Chinese hospitals, we defined nurse middle managers in this study as nurse managers other than the director of the nursing department, including assistants of the nursing department and frontline head nurses.

Inclusion criteria were: (1) nurse managers with ≥1 year of management experience; (2) a bachelor's degree or higher; (3) clear verbal communication skills; (4) possessing extensive experience in patient safety or quality management; and (5) voluntary participation. Exclusion criteria were: (1) nurse managers undertaking temporary training placements from other hospitals; and (2) individuals unable to participate due to special circumstances such as health conditions. Sample size was determined according to the principle of information power, aiming to capture a diverse range of experiences relevant to the research questions (27).

### 3.3 Data collection

The semi-structured interview guide was informed by a literature review, team deliberations, and further refined following pilot interviews with two head nurses. The final interview guides were approved by the research group (Supplementary data S2).

Between June and August 2025, all interviews were conducted face-to-face with no other individuals present. Prior to each session, the researcher established rapport, explained the study objectives and obtained informed consent, assuring participants of their anonymity. Interviews were audio-recorded, with the guide applied flexibly; techniques such as active listening and probing were employed to elicit authentic narratives, while field notes captured non-verbal cues (e.g., facial expressions). The audio recordings were verbatim transcribed into textual data within 24 h after each interview by two primary researchers (JD and LD). The transcripts were returned to the participants for verification to ensure accuracy. Data collection continued until the 12th participant was interviewed, followed by two additional interviews that yielded no new insights into the research topics, at which point data sufficiency was achieved and data collection was concluded. The average duration of the interviews was approximately 35 min.

### 3.4 Data analysis

Data analysis was conducted concurrently with data collection. The interview recordings were professionally transcribed verbatim in Chinese, pseudonymized (e.g., N1) and anonymized before being imported into NVivo 12 software (QSR International Pty Ltd., Burlington, MA, Australia) by the first author (JD) for coding and data management. Reflexive thematic analysis was undertaken following Braun and Clarke's six-phase process (28, 29), which included: (1) familiarization with the transcripts and note-taking; (2) systematic coding across the dataset; (3) generating initial themes from the coded and organized data; (4) developing and reviewing themes; (5) refining, defining and naming themes; and (6) writing the analytic report, supported by illustrative verbatim quotations that captured the essence of each theme and subthemes. The two primary researchers (JD and LD) read the transcripts verbatim, capturing meaningful content at both semantic and latent levels, generating initial codes, and clustering these codes into themes representing shared meanings. All researchers collaboratively reviewed, refined and named the themes, reaching agreement on the final thematic structure and supporting quotations through discussion. To ensure semantic and cultural accuracy, themes and quotations were translated into English by the primary researcher (JD) and back-translated by another team member (LD) for verification (30).

### 3.5 Ethical considerations

Ethical approval was obtained from the Ethics Committee of Jinling Clinical Medical College of Nanjing University of Chinese Medicine (DZQH-KYLL-24-29) on August 26, 2024. All participants were fully informed about the study and the voluntary nature of their participation, including their right to withdraw at any time without adverse consequences, and provided written informed consent before enrollment. To ensure participant confidentiality, all interview data were coded and anonymized, with electronic files stored on password-protected computers and paper materials secured in locked cabinets accessible only to the principal investigator and authorized research personnel. No data were disclosed or used for any purpose beyond the study without participants' consent.

### 3.6 Rigor and reflexivity

To ensure the study's rigor, credibility, dependability, confirmability, and transferability of findings were maintained. Credibility was established by the interviewer maintaining a non-judgmental attitude and demonstrating genuine empathy throughout the interviews, which fostered a trusting relationship with participants and encouraged them to openly share their perspectives and experiences. Transferability was supported by providing rich, detailed descriptions of participants' backgrounds and contexts, enabling readers to assess the applicability of the findings to other settings. Confirmability and dependability were ensured through a comprehensive audit trail, which included systematic documentation of interview guides, audio recordings, transcripts and analytic procedures, allowing readers to trace the research process and evaluate the accuracy and justification of the findings.

All interviews were co-conducted by two female Master's candidates, where Researcher 1 (JD) facilitated the dialogue and Researcher 2 (LD) documented observational notes. Although neither was a practicing nurse manager, their clinical experience and extensive engagement with the literature on patient safety ensured a deep understanding of the research topic. Reflexivity was embedded throughout the entire research process. Specifically, during data generation, the interviewers' positions as Master's students may have influenced participants' responses—for example, nurse managers might have perceived them as outsiders, potentially leading to reticence on sensitive topics. To mitigate this potential influence, confidentiality was emphasized before each interview, ample interaction was encouraged during the sessions, and efforts were made to foster a relaxed atmosphere, thereby promoting participants' open expression. In data analysis, the researchers' professional backgrounds supported theme development and interpretation but also carried risks of over-interpretation or misinterpretation of participants' accounts. Regular team discussions therefore provided a critical space for examining emerging themes from diverse professional viewpoints, leading to a richer interpretation of meanings.

## 4 Results

### 4.1 Characteristics of participants

The study included 14 participants (13 females, one male) with a mean age of 39.21 years (range: 29–47 years). In terms of education level, 12 participants held a bachelor's degree, and two held a master's degree. The participants had an average of 18.21 years of hospital work experience (range: 4–30 years) and 6.93 years of nursing management experience (range: 1–20 years). Nine participants held intermediate professional titles, and five held senior professional titles. Participant characteristics are shown in Table 1.

**Table 1:** The demographic characteristics of Participants (*N* = 14).

Characteristics	Participants (*N* = 14)
Age (years), mean (SD)	39.21 (5.29)
Work experience (years), mean (SD)	18.21 (6.82)
Management experience (years), mean (SD)	6.93 (5.65)
Gender, *n* (%)	
Male	1 (7.14%)
Female	13 (92.86%)
Educational background, *n* (%)	
Bachelor	12 (85.71%)
Master	2 (14.29%)
Professional title, *n* (%)	
Intermediate	9 (64.29%)
Senior	5 (35.71%)
Department, *n* (%)	
Nursing office	2 (14.29%)
Radiotherapy	1 (7.14%)
Neurology	1 (7.14%)
Oncology	1 (7.14%)
Reproductive medicine	1 (7.14%)
Hepatology	1 (7.14%)
Infectious diseases	1 (7.14%)
Intensive care unit	1 (7.14%)
Orthopedics	1 (7.14%)
Emergency medicine	1 (7.14%)
Cardiothoracic surgery	1 (7.14%)
Pediatrics	1 (7.14%)
Cardiology	1 (7.14%)

### 4.2 Summary of findings

**Figure 1:**
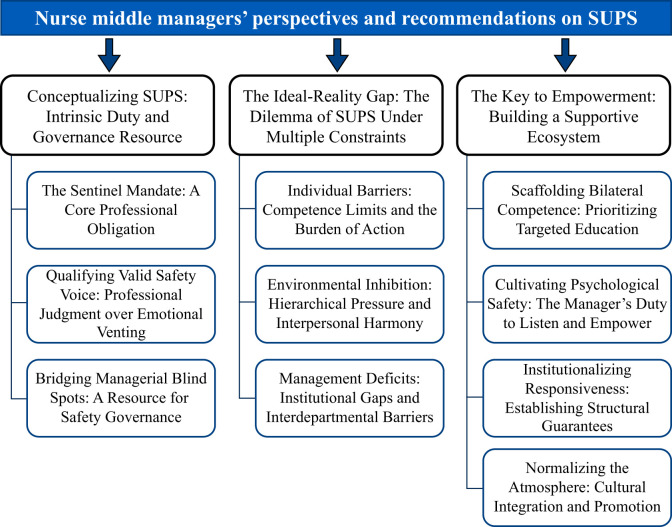
Thematic map.

Three themes and 10 subthemes (Figure 1) were generated to reflect nurse managers' perceptions and recommendations regarding nurses' SUPS: (1) Conceptualizing SUPS: Intrinsic Duty and Governance Resource; (2) the Ideal-Reality Gap: The Dilemma of SUPS Under Multiple Constraints; and (3) the Key to Empowerment: Building a Supportive Ecosystem. These themes and subthemes are discussed in detail below with illustrative supporting quotes (Supplementary data S3).

### 4.3 Theme 1: conceptualizing SUPS: Intrinsic Duty and Governance Resource

This theme delineates how nurse managers conceptualized the nature of SUPS. Rather than viewing it merely as discretionary “extra-role” behavior, participants constructed SUPS as a core component of the nurse's professional identity. This construction involves a dual perspective: ethically, it is framed as a non-negotiable obligation for patient protection; practically, it is valued as a strategic resource that managers rely upon to navigate clinical complexities and mitigate governance blind spots.

#### 4.3.1 The sentinel mandate: a core professional obligation

Nurses serve as sentinels at the clinical frontline. Participants unanimously agreed that, due to their frequent and direct contact with patients, nurses play an irreplaceable role as a critical line of defense in intercepting errors and eliminating hazards.

*Nurses themselves are a crucial line of defense for patient safety*… *although not always the primary one, they play a key role in managing details or preventing avoidable errors. Being the role with the most frequent patient contact, they are often the primary person responsible. (N10)*

Anchored in this professional positioning, participants viewed SUPS as an ethical imperative. As the final executors of care delivery, nurses bear ultimate responsibility for patient safety; thus, voicing safety hazards constitutes an intrinsic professional requirement.

*As frontline clinical staff*… *speaking up for patient safety is crucial; it is every nurse's responsibility and mandatory duty. (N1)*

*If such safety hazards occur, nurses have the obligation to actively point out and report them. This is an act of responsibility toward oneself and the patient. (N14)*

#### 4.3.2 Qualifying valid safety voice: professional judgment over emotional venting

Participants perceived effective safety voice not merely as speaking up, but as a proactive practice driven by benevolence and constructiveness, aimed at risk identification and problem-solving. This distinguishes SUPS fundamentally from emotional venting or accusatory complaints.

*Good safety voice should be a benevolent act*… *worth encouraging them to discover and solve problems*… *but simply complaining about issues is not constructive voice. (N1)*

Therefore, the manner of expression is critical; participants expected voice to be objective and non-accusatory. Furthermore, they expected nurses to possess a holistic perspective, transcending simple order execution to exercise critical thinking regarding potential risks or systemic vulnerabilities.

*The impact of speaking up is positive, but it depends on the expression. It cannot be accusatory; it must be based on the problem itself rather than on personal emotions. (N10)*

*As a nurse, my thinking should not just stop at the immediate problem. Instead, I should be able to step back, possess a certain level of critical thinking, and judge what consequences might arise from this event. (N11)*

#### 4.3.3 Bridging managerial blind spots: a resource for safety governance

Participants acknowledged the objective limitations of their oversight due to functional divisions. They viewed nurses' SUPS as a necessary compensatory mechanism to illuminate operational blind spots. Relying on the frontline perspective directly alleviates the managerial burden of monitoring clinical details.

*After all, nurses are the ones in direct contact with patients. Often, as head nurses, we cannot be at the bedside every day; we rely more on nurses to identify problems at every stage. (N5)*

*Nurses' active participation in safety management definitely reduces my burden*…*Details I might not have considered in the past can now receive more feedback. (N2)*

Based on this complementarity, managers framed SUPS as a critical pillar for optimizing workflows and enhancing nursing quality. They highlighted that the refinement of ward standards and institutional processes relies heavily on the constructive insights provided by frontline staff.

*Through their expression and feedback, the department can adjust and optimize workflows in a timely manner to ensure patient safety. (N1)*

*Regarding the standardized ward construction*… *or workflow revisions, many improvements are inseparable from nurses' daily suggestions…such speaking up is instrumental for detail optimization and workflow perfection. (N10)*

### 4.4 Theme 2: the ideal-reality gap: the dilemma of SUPS under multiple constraints

While participants unanimously affirmed the intrinsic value of SUPS, they acknowledged a stark contrast between this professional ideal and the reality of clinical practice. The current status of speaking up is characterized by a predominance of “silence over voice” and “remedy over prevention.” Managers observed that most nurses tend to adopt a reactive mindset, providing feedback only after adverse events have occurred, rather than proactively identifying latent risks. This lag in response undermines the early warning function that safety voice is intended to serve.

*It's not particularly common. We have 24 nurses; besides me*… *I estimate maybe two-fifths would speak up or frequently provide feedback*… *(N9)*

*In comparison, some nurses just complete their tasks. As long as no issues arise, they feel everything is fine and won't proactively identify potential risks and hazards. (N7)*

*Most nurses don't think preventively; they wait until something happens before taking action*…*they won't proactively propose constructive suggestions like “how should we avoid these problems.” (N8)*

This paradox reflects an implicit dilemma stemming from the conflict between professional expectations and the multiple constraints of the real-world environment. From individual apprehensions to institutional deficiencies, intertwined resistances hinder the normalization of voice behavior.

#### 4.4.1 Individual barriers: competence limits and the burden of action

Why are nurses reluctant to speak up? Participants identified that individual-level barriers primarily stemmed from limitations in professional competence. Some nurses lacked the professional acuity to recognize latent hazards or the communication proficiency to articulate issues clearly, rendering them “willing but unable” to speak up. Other nurses lacked professional dedication, viewing safety concerns as disconnected from their personal responsibilities.

*The root cause is often that nurses are unaware of the safety hazard*… *tracing it back, they likely did not realize it was a safety issue. (N5)*

*Often, when nurses speak up, they may express themselves unclearly; what they intend to say and what is actually conveyed may differ. (N6)*

*It also relates to nurses' personal identification with or engagement in their work. Some nurses have the attitude of “the less trouble the better,” possibly feeling that unit matters are not part of their personal career aspirations. (N2)*

Beyond competence factors, a deeper impediment lies in nurses' realistic consideration of personal costs. Managers pointed out a phenomenon in clinical work where the act of speaking up is often tethered to the subsequent burden of problem-solving. This expectation leads nurses to fear that voicing concerns will result in additional work duties, prompting them to choose avoidance as a form of self-protection.

…*If they make a suggestion, it might mean they have to spend more time helping to implement it, so some nurses are reluctant to do so. (N8)*

*Many nurses feel this doesn't really concern them, or they worry–if I raise this, management might adopt it, and that could mean extra work for me, right? Since suggesting things doesn't benefit them and might even create more work, they just stay quiet. (N10)*

Additionally, nurses tended to face the dilemma of having to prioritize immediate routine duties over the extra labor of SUPS, due to time and energy constraints.

*There's also the workload pressure. Nurses are already very busy with their daily tasks, so taking on anything extra can feel overwhelming–they often simply avoid it. (N8)*

#### 4.4.2 Environmental inhibition: hierarchical pressure and interpersonal harmony

The restrictive organizational climate within clinical units inhibits voice behavior. Participants acknowledged that authoritarian leadership suppresses nurses' willingness to speak up, while hierarchical power distance creates an invisible pressure, deterring them from expressing genuine opinions to their superiors.

*One must never be like some autocratic head nurses who have a “my word is law” attitude. If that's the case, staff definitely won't want to say anything*… *they just follow whatever you say and stop speaking up proactively. (N6)*

…*From a nurse's perspective, there remains a sense of fear–they hesitate to approach leaders. Like when I was a staff nurse, I also avoided speaking with managers out of that fear, that intangible pressure. (N9)*

Simultaneously, the cultural emphasis on harmony introduces a complex horizontal constraint. within this collectivist context, nurses worry that voicing problems publicly might be construed by peers as disrupting team unity or triggering interpersonal conflict. This concern compels them to prioritize relational relationships over patient safety.

*If collegial relationships are fragile, even if a nurse identifies a problem, may dare not raise it directly out of fear that it might damage her relationship with colleagues. (N7)*

*They might worry about being seen as “telling on” others–well, it's about the relationship between coworkers, and it could make things really unpleasant. So that is a concern they have*… *(N14)*

#### 4.4.3 Management deficits: institutional gaps and interdepartmental barriers

The absence of responsive reporting channels transforms speaking up from a professional duty into a futile endeavor. When repeated proposals go unanswered, nurses were forced into silence, fearing that persistent advocacy will be dismissed as merely being troublesome.

*Even if nurses have constructive suggestions, there are no effective channels to report them*… *(N7)*

*Sometimes as a manager, I am reluctant to raise issues repeatedly. I feel like, ‘Look, I've raised this so many times and it's still not resolved'; people will just start thinking you are being a nuisance. (N5)*

Existing mechanisms to support or encourage SUPS are scarce. Current systems primarily focus on adverse event reporting, yet the cumbersome reporting procedures create a significant burden for nurses.

*Our unit currently lacks specific incentive mechanisms*… *so nurses feel that if their suggestions aren't taken up, it's simply not worth speaking up. (N7)*

*The hospital has no specific policy to encourage it…Beyond handling adverse events, after reporting, nurses are required to complete various forms and documentation*… *the process is quite complex. (N10)*

In addition, speaking up may prove ineffective when safety solutions necessitate cross-departmental coordination. Participants described a profound sense of powerlessness, as issues perceived as critical by nursing staff are often regarded as non-priorities by other departments, leaving hazards unresolved.

*Some problems that seem urgent to nursing may not be a priority for other departments… for instance, I've recommended updating old equipment, but that doesn't necessarily lead to any action. (N5)*

*In outpatient settings, issues such as equipment problems or environmental hygiene… are recognized as real concerns. Yet when reported to the relevant departments with no active response or solution… it leaves everyone feeling that speaking up is futile–simply a waste of time. (N13)*

### 4.5 Theme 3: the key to empowerment: building a supportive ecosystem

This theme synthesizes solutions proposed by nurse managers to overcome current barriers to voice behavior. Interview data indicated that managers considered verbal encouragement alone insufficient to bridge the knowing-doing gap. Participants thus advocated for a multifaceted approach involving targeted education, managerial support, institutional safeguards, and a supportive culture, aiming to foster an environment where nurses feel able, safe, and motivated to speak up.

#### 4.5.1 Scaffolding bilateral competence: prioritizing targeted education

Most participants pointed out the lack of specific curricula focused on SUPS. This gap results in nurses lacking the necessary cognitive awareness, risk identification skills, and assertive communication competence to engage proactively in safety practices.

*Currently, our hospital does not seem to have specialized training for safety voice*… *although there is case sharing for adverse events, it does not specifically revolve around how to raise safety concerns. (N8)*

To bolster nurses' self-efficacy, participants recommended training that moves beyond theory to include the value of speaking up and practical communication skills.

*First, we must let everyone understand the importance*… *through specialized training. We can use specific examples to illustrate where hazards can be found and then what measures to take. (N7)*

*Training should not only improve theoretical and communication skills but also include value orientation. We need to tell nurses why safety voice is critical for patients and the unit. (N8)*

Notably, safety dialogue is not a unilateral act of speaking but a dynamic interaction. As the primary recipients of SUPS, managers equally require training to update their paradigms and master the competencies needed to facilitate and respond to voice, thereby effectively mobilizing the team.

*If I, as a manager, lack sufficient understanding*… *I cannot guide nurses to express their views*… *If the manager does not value this concept, how can they mobilize the entire team*…*? (N11)*

*The hospital should provide guidance for head nurses and team leaders*… *specifically on how to effectively respond to suggestions*… *providing training on communication and management skills is necessary to improve efficiency and teamwork. (N10)*

#### 4.5.2 Cultivating psychological safety: the manager's duty to listen and empower

To address nurses' psychological hesitation to speak up, participants unanimously emphasized that leadership attitude and behavior are crucial in breaking the silence. Team managers must demonstrate an open and inclusive leadership style in daily work, patiently listening rather than hastily dismissing concerns, thereby creating a psychologically safe space for expression.

*As a manager, you can't be impatient with everything they say… You have to listen patiently, because important issues might surface even from minor talk. (N5)*

…*we need to create an environment where nurses dare to speak up and are encouraged to express their views… and we also need to guide their thinking. (N9)*

In practice, participants highlighted the importance of empowering staff through participatory management to build ownership and confidence. Leading by example was also seen as critical–managers must visibly model safety behaviors to set a clear standard for the team.

*Managers need to empower nurses*… *When nurses are granted the right to participate*… *they are no longer mere followers but key members*… *Empowerment helps them view issues from a holistic perspective*… *This sense of participation enhances confidence, motivating them to provide more valuable safety voice. (N8)*

…*the manager's own safety literacy has a huge impact*… *We must firmly hold the safety baseline*… *and use our actual actions to influence every nurse, making safety awareness a team habit. (N10)*

#### 4.5.3 Institutionalizing responsiveness: establishing structural guarantees

To counteract the sense of futility regarding speaking up, participants urged the establishment of explicit protocols with defined responsibilities to institutionally safeguard speakers.

*The institutional aspect is crucial*… *there should be a clear voice policy with distinct responsibilities*… *We must have a concrete, actionable plan. Every step should have a designated professional*… *rather than just being an empty title. (N7)*

Participants also called for expanding reporting channels and improving feedback mechanisms, such as anonymous or digital platforms, to ensure timely and transparent follow-up.

*The hospital could set up dedicated channels, like a dean's suggestion box or anonymous feedback… This allows issues to be resolved harmoniously without straining relationships. (N10)*

*If there were an information platform where everyone could see the status*… *that would make it much more open and transparent. (N4)*

Furthermore, participants proposed integrating SUPS into performance appraisals, ensuring that constructive suggestions receive substantive responses and tangible rewards, thereby establishing a closed loop of positive reinforcement.

…*consider incorporating constructive suggestions into unit performance evaluations*… *For nurses who frequently raise valid suggestions, we could give appropriate preference in year-end evaluations or external study opportunities. (N2)*

#### 4.5.4 Normalizing the atmosphere: cultural integration and promotion

Beyond institutional guarantees and training, participants proposed utilizing diversified promotional channels to foster an open and trusting climate, thereby embedding SUPS into the team culture.

*The hospital needs to use culture as a guide, for instance, by launching awareness activities or a Speak Up for Safety Day to spread the idea of safety voice. (N8)*

*We can also promote it through static materials. For example, in unit corridors, we can set up safety bulletin boards to display suggestion cases or relevant safety knowledge. (N6)*

Additionally, participants proposed demonstrating the SUPS process through interactive skits, helping nurses intuitively grasp its significance and value.

*Through skits, short plays, or similar dramatizations, we can show how problems are identified and risks are prevented. This helps everyone grasp the significance of safety voice more intuitively. (N8)*

## 5 Discussion

Focusing on nurse middle managers as key informants, this study explored their perceptions, realistic challenges, and strategic conceptualizations of SUPS. Three themes were identified, revealing a pronounced “ideal-reality gap.” While managers cognitively framed SUPS as a non-negotiable professional obligation (Theme 1), the enactment of this behavior in clinical practice is constrained by individual competence deficits, hierarchical silence, and systemic futility (Theme 2). This passive state is characterized by “more silence than speaking up” and “more remediation than prevention,” aligns with a study in South Korea (22), which noted that current speaking-up practices are often limited to post-hoc reporting of adverse events and lack an organizational environment that encourages proactive, open communication. Consequently, our findings suggest that fostering effective SUPS requires a paradigm shift: moving the locus of responsibility from individual nurse assertiveness to the construction of a multi-dimensional supportive ecosystem (Theme 3). To systematically analyze this stagnation and explore potential solutions, this study applied the Social-Ecological Systems Theory (SET) (31) as an analytical framework. The following discussion integrates these findings across micro, meso, and macro levels to propose a holistic intervention blueprint for SUPS.

### 5.1 Implementing training to enhance cognitive awareness of SUPS among nurses and managers

At the micro level, our findings highlight that nurses are prone to silence regarding safety hazards or unprofessional behaviors if they lack sufficient recognition of the value of SUPS or lack necessary communication skills. This finding corroborates the study by Lee et al. (14), which identified individual assertiveness, professional self-efficacy, and the perceived importance of speaking up as key determinants of nurses' willingness to advocate for patient safety. Furthermore, SUPS is not merely a unidirectional act by nurses but a bidirectional interaction. Research indicates that leader voice solicitation conveys positive signals that encourage expression and enhances the willingness of employees to participate in improvements (32). Conversely, if managers lack a correct understanding of the value of SUPS and misinterpret it as challenging authority, they may unintentionally stifle the motivation of nurses to speak up (6, 33). Participants in this study also recognized the necessity of strengthening guidance and feedback regarding SUPS in daily work, underscoring the urgency of enhancing shared cognition between management and frontline nurses.

From the perspective of SET, SUPS is not an innate instinct but a professional competence requiring systematic education. The identification of safety hazards is a prerequisite for speaking up. Participants reported that nurses with critical thinking and risk recognition skills were more capable of detecting potential safety concerns. Furthermore, existing literature confirms the effectiveness of speaking-up training, such as the high-fidelity simulations by Guris et al. (34), which significantly improved the safety communication efficacy of new employees. Participants in this study noted that current hospital training systems predominantly focus on clinical skills and nurse-patient communication, lacking specific education on SUPS. Therefore, beyond providing general patient safety education to sharpen nurses' vigilance, nurse managers should develop systematic and scientific SUPS training programs drawing on international experience while considering the cultural context and clinical reality of China.

First, regarding cognitive reshaping, methods such as role-playing and scenario simulation should be used to reinforce the sense of responsibility of nurses as safety sentinels, helping them transcend a narrow focus on their own duties to establish a systemic view of safety. Second, regarding skill empowerment, structured communication tools such as SBAR, CUS, and the Two-Challenge Rule should be incorporated into mandatory curricula to help nurses confidently express safety concerns in complex clinical situations (35). Meanwhile, our findings emphasize the need for targeted training for nurse managers to master skills in effective listening, scientific guidance, and active response to SUPS. As advocated by Barlow et al. (36), SUPS needs to shift from the traditional “singular act” of speaking up to a safety negotiation characterized by “shared voice” and equal participation between the speaker and receiver. Such bidirectional, practical training enhances shared cognitive awareness, facilitating the effective translation of SUPS from knowledge to action.

### 5.2 Emphasizing managerial guidance in fostering a psychological safety climate

At the meso level, as the core of the team, the behavioral paradigms and value orientations of managers exert a direct and significant influence on employee behavior (15). Even when nurses have formed an intention to speak up, the translation of this intention into actual behavior may be moderated by multiple factors (16). Our study further found that potential additional workloads following voice behavior, or experiences of suggestion failure due to ineffective interdepartmental coordination, can weaken nurses' assessment of the usefulness and safety of speaking up, thereby reinforcing a “status quo” attitude. In this process, the role of managers is particularly critical. High-quality leader-member exchange relationships can effectively reduce power distance, making nurses perceive communication with superiors as smoother and less risky, while strengthening their confidence that leaders will respond seriously and address issues (37, 38). Therefore, to address nurses' reluctance to speak up, interventions should focus not only on enhancing their competencies and professional dedication, but also on encouraging leaders to critically reflect on the impact of their own behavior and implement proactive, effective management practices to improve psychological safety climate.

In this study, participants further emphasized that managers need to maintain an open and inclusive leadership style, actively listen to the opinions of nurses, and create a supportive environment. These elements align with previous research (17), which indicates that managers should adeptly use inclusive language, actively solicit suggestions and concerns by asking questions like “What do you think?” and provide appropriate feedback such as “Thank you for your suggestion.” These actions enhance familiarity and trust between managers and staff. Furthermore, participants generally believed that managers possessing excellent safety skills, concepts, and awareness can more effectively transmit high-quality safety philosophies and behaviors, thereby reinforcing the team safety climate. Alingh et al. (39) similarly found that commitment-based safety management by nurse managers helps enhance team psychological safety, motivating nurses to proactively voice safety concerns. Therefore, nurse managers must enhance their safety competencies and lead by example through active engagement in training and strict regulatory compliance. Simultaneously, they should explicitly articulate support for SUPS and promote the safety vision, translating their commitment into visible behavioral benchmarks.

Appropriate empowerment was also identified by participants as a crucial strategy to stimulate the intrinsic motivation of nurses for safety. Consistent with Hashemian et al. (38), this study emphasizes that empowering nurses within the institutional framework and recognizing their role in decision-making can improve overall nursing quality and enhance their sense of belonging in the healthcare system. This suggests that nurse managers can draw on the Magnet nursing management model to reasonably delegate authority and empower nurses to participate in safety management and decision-making. This approach fosters a respectful and trusting leader-member relationship, thereby eliminating the psychological concerns of nurses (40). Additionally, addressing the issue of insufficient SUPS participation due to heavy workloads mentioned by participants, nurse managers should fully consider indicators such as workload, technical difficulty, and nursing risks. Implementing flexible scheduling and dynamic staffing can reserve safety observation time for nurses to identify and report hazards.

### 5.3 Improving the SUPS support system and shaping a positive safety culture

At the macro level, institutional deficiencies and barriers to cross-departmental collaboration constitute critical structural obstacles hindering SUPS. Consistent with Umoren et al. (33), who found that the lack of specific and feasible institutional support hinders speaking up, this study confirms that ambiguous regulations leave nurses without robust policy support when confronting safety hazards. In particular, this study revealed the implicit workplace rule dictating that whoever raises an issue must resolve it. This practice forcibly binds problem identification with problem resolution, causing nurses to choose silence for self-protection.

From the perspective of SET, the core function of the macro system is to provide solid safety boundaries for individual behaviors. Therefore, hospital management must establish regulations with clear rights and responsibilities. We recommend establishing a dedicated committee to ensure that the rights of those who speak up are protected, thereby eliminating concerns at the institutional level. Furthermore, priority should be given to establishing non-punitive policies (12, 38), broadening reporting channels, and providing anonymous reporting mechanisms. On one hand, anonymous digital platforms such as electronic suggestion boxes or mobile reporting applications can be utilized to alleviate interpersonal pressure on nurses. On the other hand, normalized communication channels should be established through safety seminars or one-on-one interviews to foster an atmosphere of open and equal dialogue. Moreover, participants emphasized that delayed or absent feedback undermines nurses' motivation to speak up by creating a sense of futility. To counter this, the study proposes a support system featuring closed-loop feedback and diversified incentives. Specific measures include assigning dedicated staff to manage reporting platforms for timely response, and maintaining a SUPS registry to regularly share progress on common issues–making feedback visible and reinforcing nurses' sense of value. To boost engagement, a blended incentive model combining material and non-material rewards is recommended. Examples include Safety Month awards, point-based systems, and linking safety reporting to performance reviews, career development, and promotion criteria, thereby strengthening SUPS behavior through positive reinforcement.

Notably, previous studies have shown that positive collaborative relationships can enhance nurses' willingness to speak up (41). However, this study suggests that interpersonal relationships may exert a “double-edged sword” effect. This phenomenon may be influenced by the Confucian principle of “harmony as paramount,” advocated by the doctrine of the mean. Nurses often worry that speaking up directly about issues might disrupt team harmony or lead to them being perceived as “troublemakers,” thus tending to avoid directly pointing out safety hazards. This finding reveals a potential inherent tension between surface harmony and deep-seated safety concerns within the Chinese cultural context. Compared to Western research that emphasizes the open, fair, and just logic of a “just culture” (42), the “harmony” constraint in the Chinese context places greater emphasis on maintaining the smooth operation of group relationships. This cultural characteristic may lead nurses to adopt more cautious strategic choices when engaging in safety voice. Therefore, in organizational culture development, hospitals should guide healthcare professionals to recognize that proactively reporting safety hazards is not intended to damage interpersonal relationships or provoke conflict. On the contrary, it is a collective effort to safeguard patients' wellbeing and also builds a protective barrier for healthcare professionals' own practice safety. Hospitals should actively foster a non-punitive patient safety management culture that promotes learning from errors. Furthermore, this study extends the perspective of Jeong and Kim (22) on achieving normalized promotion of SUPS through announcements and information sharing within reporting systems. Participants supplemented this with specific environmental intervention strategies, noting that physical spaces should be fully utilized for visual education. For instance, setting up safety bulletin boards in ward corridors to display excellent SUPS cases allows safety awareness to be subtly integrated into daily work scenarios. Meanwhile, to strengthen the SUPS climate, participants recommended thematic activities featuring nurse-led experience and story sharing. In summary, our findings indicate that hospital departments should utilize diverse forms of publicity and interaction to establish a team consensus of zero tolerance for silence on safety issues.

### 5.4 Strengths and limitations

Set within the Chinese healthcare context, our research explores nurse middle managers' perspectives on nurses' SUPS, offering valuable insights for its promotion. Methodological rigor was ensured through a pilot study prior to data collection. Furthermore, applying SET throughout the discussion facilitated the synthesis of identified barriers and management strategies, clarifying specific targets for practice and policy change. However, several limitations should be acknowledged. First, although purposive sampling was used to ensure participant diversity, recruitment from a single hospital may restrict the transferability and applicability of the findings. Second, only one participant was male; while this reflects the gender demographics of the nursing profession, it potentially limits the representation of male perspectives. Additionally, the reliance on a single data source may introduce attribution bias. Future research could incorporate multi-informant perspectives, such as those of frontline clinical nurses, to further validate and enrich the conclusions.

### 5.5 Recommendations for further research

Overall, the findings underline several avenues for future inquiries. First, future research should contrast the views of diverse healthcare professionals with those of nurse managers to reveal the mechanisms of the cognition-practice gap. Second, to develop effective promotion strategies such as leadership interventions or institutional optimization, engaging stakeholders to co-design supportive schemes is vital. Lastly, investigating SUPS practice variations across different hospital levels, regions, cultures, and departments will deepen our multi-dimensional understanding of its application.

### 5.6 Implications for policy and practice

This study's findings highlight the importance of multi-level strategies to promote SUPS in clinical practice. Specifically, institutions should provide systematic opportunities for nurse managers and nurses to develop SUPS competencies. Furthermore, hospital policies should explicitly integrate SUPS as a core patient safety requirement, supported by non-punitive reporting, timely feedback, and resource allocation. At the practice level, nurse managers must foster an inclusive atmosphere, optimize staffing, and implement empowerment and positive incentives to transform SUPS into a normalized team behavior.

## 6 Conclusion

This study explored the perceptions and support strategies of nurse managers regarding SUPS through in-depth interviews. Participants universally recognized the composite value of SUPS as a professional obligation and a proactive safety practice. However, they indicated that current dilemmas stem primarily from insufficient individual competence, interpersonal and workload pressures, and the absence of institutional and feedback mechanisms. To address these challenges, this study emphasizes the necessity of constructing a supportive ecosystem encompassing targeted education, leadership modeling, institutional guarantees, and cultural cultivation. From a management perspective, these findings offer vital localized insights for understanding and promoting SUPS in healthcare institutions, laying a theoretical foundation for developing multi-level, structured interventions.

## Data Availability

The original contributions presented in the study are included in the article/Supplementary material, further inquiries can be directed to the corresponding author.
