# Supplementary material for: The ideal-reality gap: a qualitative study of nurse middle managers' perspectives on speaking up for patient safety
Source: Front Public Health. 2026 Apr 9;14:1790424. doi: 10.3389/fpubh.2026.1790424 (PMC13102841; doi:10.3389/fpubh.2026.1790424)
Supplement: Supplementary file 1 [file Supplementary_file_1.pdf]

Supplementary Material

1 Supplementary Data 1

COREQ (COnsolidated criteria for REporting Qualitative research) Checklist

A checklist of items that should be included in reports of qualitative research. You must report the page number in your manuscript where you consider each of the items listed in this checklist. If you have not included this information, either revise your manuscript accordingly before submitting or note N/A.

Topic	Item No.	Guide Questions/Description	Reported on Page No.
**Domain 1: Research team and reflexivity**			
Personal characteristics			
Interviewer/facilitator	1	Which author/s conducted the interview or focus group?	Page 03
Credentials	2	What were the researcher's credentials? E.g. PhD, MD	Page 02
Occupation	3	What was their occupation at the time of the study?	Page 02
Gender	4	Was the researcher male or female?	Page 03
Experience and training	5	What experience or training did the researcher have?	Page 02
Relationship with participants			
Relationship established	6	Was a relationship established prior to study commencement?	Page 03

Topic	Item No.	Guide Questions/Description	Reported on Page No.
Participant knowledge of the interviewer	7	What did the participants know about the researcher? e.g. personal goals, reasons for doing the research	Page 03
Interviewer characteristics	8	What characteristics were reported about the interviewer / facilitator? e.g. Bias, assumptions, reasons and interests in the research topic	Page 04
**Domain 2: Study design**			
Theoretical framework			
Methodological orientation and Theory	9	What methodological orientation was stated to underpin the study? e.g. grounded theory, discourse analysis, ethnography, phenomenology, content analysis	Page 03
Participant selection			
Sampling	10	How were participants selected? e.g. purposive, convenience, consecutive, snowball	Page 02
Method of approach	11	How were participants approached? e.g. face–to–face, telephone, mail, email	Page 03
Sample size	12	How many participants were in the study?	Page 04
Non–participation	13	How many people refused to participate or dropped out? Reasons?	N/A
Setting			

Topic	Item No.	Guide Questions/Description	Reported on Page No.
Setting of data collection	14	Where was the data collected? e.g. home, clinic, workplace	Page 02
Presence of non– participants	15	Was anyone else present besides the participants and researchers?	Page 03
Description of sample	16	What are the important characteristics of the sample? e.g. demographic data, date	Page 04+Table 1
Data collection			
Interview guide	17	Were questions, prompts, guides provided by the authors? Was it pilot tested?	Page 03+Data 2
Repeat interviews	18	Were repeat inter views carried out? If yes, how many?	Page 03
Audio/visual recording	19	Did the research use audio or visual recording to collect the data?	Page 03
Field notes	20	Were field notes made during and/or after the interview or focus group?	Page 03
Duration	21	What was the duration of the inter views or focus group?	Page 03
Data saturation	22	Was data saturation discussed?	Page 03
Transcripts returned	23	Were transcripts returned to participants for comment and/or correction?	Page 03
**Domain 3: analysis and findings**			
Data analysis			

Topic	Item No.	Guide Questions/Description	Reported on Page No.
Number of data coders	24	How many data coders coded the data?	Page 03
Description of the coding tree	25	Did authors provide a description of the coding tree?	Page 04+Fig.1+Data 3
Derivation of themes	26	Were themes identified in advance or derived from the data?	Page 04
Software	27	What software, if applicable, was used to manage the data?	Page 03
Participant checking	28	Did participants provide feedback on the findings?	Page 03
Reporting			
Quotations presented	29	Were participant quotations presented to illustrate the themes/findings? Was each quotation identified? e.g. participant number	Page 04-09+Data 3
Data and findings consistent	30	Was there consistency between the data presented and the findings?	Page 04-09
Clarity of major themes	31	Were major themes clearly presented in the findings?	Page 04
Clarity of minor themes	32	Is there a description of diverse cases or discussion of minor themes?	Page 04-09

Developed from: Tong A, Sainsbury P, Craig J. Consolidated criteria for reporting qualitative research (COREQ): a 32-item checklist for interviews and focus groups. *International Journal for Quality in Health Care*. 2007. Volume 19, Number 6: pp. 349 – 357

Once you have completed this checklist, please save a copy and upload it as part of your submission. DO NOT include this checklist as part of the main manuscript document. It must be uploaded as a separate file.

2 Supplementary Data 2

Interview Schedule

Part 1: Introduction

- **Welcome and Appreciation:** Express sincere gratitude to the nurse managers for participating in this interview.
- **Self-Introduction and Background:** Briefly introduce the researchers and explain the study's aim: to explore nurse managers' genuine perspectives and suggestions regarding "speaking up for patient safety" from the perspective of nursing management practice.
- **Informed Consent and Ethics:**
  - Explain that the interview will be audio-recorded, but all personal and hospital identities will be anonymised in subsequent transcription and analysis to ensure strict confidentiality.
  - Emphasize that there are no right or wrong answers; encourage the sharing of authentic experiences and feelings.
- **Demographic Data:** Collect background information, including age, educational level, work experience.

Part 2: Opening Question

Could you describe the current team climate and the status of safety management in your unit?

Main Questions:

(1) How do you conceptualise speaking up for patient safety? In your mind, what does the ideal state of speaking up look like?

Prompts:

- Do you view pointing out problems as a nurse's core responsibility or as a form of extra help (or extra-role behaviour)? Why?
- What does "speaking up for patient safety" mean to you personally?

(2) In your daily work environment, what is the actual situation of nurses' speaking up for patient safety? Does it align with your expectations?

Prompts:

- What is your perception of the climate for speaking up for patient safety in your team? Do you consider it good or suboptimal?
- Can you share a specific case from your experience that left a deep impression—either where a nurse successfully spoke up or remained silent? What was the context at that time?

(3) Based on your experience and observation, what factors hinder nurses from speaking up for patient safety freely?

Prompts:

- What role do interpersonal dynamics or unit culture (e.g., interaction with doctors or senior nurses) play in this?
- Based on your observation, what specific concerns or worries might nurses have?

(4) How do you typically react when a nurse raises safety concerns or suggestions? What is your personal experience in managing these situations?

Prompts:

- Have you encountered specific challenging situations when responding to or supporting nurses? What were your personal feelings at that time?

(5) Have you provided any specific support to promote nurses speaking up for patient safety? What specific methods have you used?

Prompts:

- How do you perceive the effectiveness of these methods?

(6) Looking forward, what measures do you think are needed to better promote nurses speaking up for patient safety? Please provide examples.

Prompts:

- Based on the barriers you mentioned, do you have any ideas on how best to support nurses speaking up for patient safety?
- If the hospital were to develop a "Supportive Programme for Speaking Up for Patient Safety," what key components do you think must be included?

Part 3: End

- Ask if the participants has any additional information or thoughts to add.
- Mention that if participants have any questions, they can always contact us.
- Reiterate thanks for their participation.

3 Supplementary Data 3

Theme, sub-theme and quotes

Themes	Sub-themes	Quotes
Theme 1: Conceptualizing SUPS: Intrinsic Duty and Governance Resource	The Sentinel Mandate: A Core Professional Obligation	• *Nurses act as sentinels in clinical work. Their suggestions can effectively intercept or prevent many errors and incidents. (N6)*<br>• *I think it's very important because nurses are the primary personnel directly in contact with patients in clinical work. Personally, I believe nurses have a strong obligation to speak up for patient safety. If nurses can not only identify problems but also provide constructive suggestions at a deeper level to us nurse managers, we would be even more pleased to see that. (N13)*<br>• *If such safety hazards occur, nurses have the obligation to actively point out and report them. This is an act of responsibility toward oneself and the patient. (N14)*<br>• *It's for patient safety, really. As a nurse, professional ethics are of course the most basic. Clinical nurses are on the front lines; they likely have a clearer understanding of the patient's condition and any potential risks. They observe directly. Some nurses can spot issues in time and then take preventive measures. So, the main consideration behind this is really nursing safety. (N4)*<br>• *I think this is one of the most fundamental qualities and requirements for a nurse involved in unit management. (N10)*<br>• *As frontline clinical staff... speaking up for patient safety is crucial; it is every nurse's responsibility and mandatory duty. (N1)*<br>• *Nurses themselves are a crucial line of defense for patient safety... although not always the primary one, they play a key role in managing details or preventing avoidable errors. Being the role with the most frequent patient contact, they are often the primary person responsible. (N10)*<br>• *As key frontline roles in clinical settings, nurses are the first line of defense for patient safety. (N2)*<br>• *Nurses on the clinical frontline are definitely the primary responsible personnel for patient safety risks. Additionally, nurses are also the final executors of various management systems, especially safety and core management protocols. Therefore, they are the most likely to identify problems and also the most capable of proposing effective improvement measures. (N3)*<br>• *We nurses serve as the primary responsible persons for patients. In this process, when nurses speak up for patient safety with suggestions, it essentially serves to remind us all to pay more attention to patient health and safety. (N9)*
	Qualifying Valid Safety Voice: Professional Judgment over Emotional Venting	• *My understanding of this concept is that during clinical work, when nurses identify factors or potential risks that could affect patient safety, they provide feedback or discuss them with us nurse managers or other colleagues in the department. (N13)*<br>• *I absolutely support and encourage such behavior. If a nurse is willing to do this, I would actually consider her a potential candidate for nursing management development. (N5)*<br>• *Through speaking up, nurses can demonstrate not only their professional competence but also their initiative—whether in proactive service or in actively identifying clinical issues. (N5)*<br>• *Speaking up for patient safety is probably a specific aspect of nurse voice behavior, emphasizing that nurses should voice their opinions and suggestions specifically regarding patient safety. (N4)*<br>• *I believe it manifests in everyone's daily work. When I spot a problem, I speak up. For example, if a doctor prescribes*

Themes	Sub-themes	Quotes
		the wrong order and a nurse notices it, she will point it out promptly. (N1)<br>• *As a nurse, my thinking should not just stop at the immediate problem. Instead, I should be able to step back, possess a certain level of critical thinking, and judge what consequences might arise from this event. (N11)*<br>• *If nurses can perform every task and serve patients with proactive thinking and a critical mindset, this behavior itself helps uncover potential safety hazards. That is definitely worth encouraging. (N5)*<br>• *My understanding is that nurses can proactively identify unsafe events in clinical practice, point them out promptly, and intervene, thereby reducing the occurrence of unsafe clinical events and mitigating or intercepting adverse events. (N11)*<br>• *During work, when healthcare staff observe potential hazards that may affect patient safety, they actively offer suggestions and opinions to ensure patient safety. (N9)*<br>• *Good safety voice should be a benevolent act... worth encouraging them to discover and solve problems... but simply complaining about issues is not constructive voice.(N1)*<br>• *The impact of speaking up is positive, but it depends on the expression. It cannot be accusatory; it must be based on the problem itself rather than on personal emotions. (N10)*<br>• *In my view, mere complaining and venting do not constitute speaking up. (N6)*
	Bridging Managerial Blind Spots: A Resource for Safety Governance	• *For nurses themselves, it gives them peace of mind during their shifts. Once a safety hazard is pointed out by anyone, we can act quickly to avoid it. Colleagues can supervise each other, helping everyone work more safely. (N1)*<br>• *I hope that through everyone's input, I can identify some potential issues. I often say, an individual's capacity is limited; the collective strength is the greatest. For instance, others might offer perspectives from different angles, which could bring me new insights. (N2)*<br>• *Safety voice can enhance the overall work quality of the department, ensure patient safety, and also safeguard the hospital. (N8)*<br>• *Compared to nurses who just do their work without speaking up, leaders naturally tend to assign important tasks to those who frequently voice their opinions and make suggestions. (N6)*<br>• *Through their observations and feedback, they can play a role in identifying and filling gaps in clinical practice, uncovering potential safety hazards that we managers or others might overlook, thereby effectively reducing unsafe incidents. (N2)*<br>• *The ability of nurses to identify and raise issues certainly helps the department establish a more robust safety baseline. Many specialized safety protocols in various departments actually originate from problems pointed out by nurses. (N3)*<br>• *Speaking up can hone nurses' communication skills and their ability to participate in departmental safety management. During the problem-solving process, they also gain experience and lessons. (N10)*<br>• *Nurses' active participation in safety management definitely reduces my burden…Details I might not have considered in the past can now receive more feedback. (N2)*<br>• *After all, nurses are the ones in direct contact with patients. Often, as head nurses, we cannot be at the bedside every day; we rely more on nurses to identify problems at every stage. (N5)*<br>• *Through their expression and feedback, the department can adjust and optimize workflows in a timely manner to ensure patient safety. (N1)*<br>• *Regarding the standardized ward construction... or workflow revisions, many improvements are inseparable from nurses' daily suggestions…such speaking up is instrumental for detail optimization and workflow perfection. (N10)*

Themes	Sub-themes	Quotes
		• *This is also the result of managers encouraging nurses to identify and speak up about problems early on, so nurses don't need to worry about being blamed for speaking out. It was precisely because a nurse raised this issue that I realized there are correct orders, incorrect orders, resuscitation orders, and also questionable orders in the physician's order sets. For those questionable orders, we now have a complete protocol in place. Isn't it precisely because they raised this issue that we were able to optimize our process? (N11)*
Theme 2: The Ideal-Reality Gap: The Dilemma of SUPS Under Multiple Constraints		• *In comparison, some nurses just complete their tasks. As long as no issues arise, they feel everything is fine and won't proactively identify potential risks and hazards. (N7)*<br>• *As mentioned earlier, possibly due to a lack of established mechanisms, many nurses are not very familiar with the concept of "speaking up" and don't fully understand its significance... Most nurses tend to address problems after they occur, rather than taking proactive measures before issues arise or when hidden risks haven't yet been identified. (N7)*<br>• *Currently, such behavior is not particularly widespread in clinical practice, and nurses' understanding of the concept of "speaking up for patient safety" is not deep. (N11)*<br>• *I wouldn't say it's especially common. Some nurses with stronger skills in this area might share their ideas if they have any. (N4)*<br>• *Honestly, this behavior isn't very prevalent. Based on my over ten years of clinical experience, many nurses often report issues only when they feel they can no longer keep them to themselves. (N8)*<br>• *Most nurses don't think preventively; they wait until something happens before taking action...they won't proactively propose constructive suggestions like "how should we avoid these problems". (N8)*<br>• *Typically, they don't immediately consider, "What consequences might this situation have, and how should we prevent them?" Instead, they deal with problems only after they occur. (N8)*<br>• *It's not particularly common. We have 24 nurses; besides me... I estimate maybe two-fifths would speak up or frequently provide feedback... (N9)*<br>• *I haven't really heard of this concept, and currently, I haven't seen it explicitly mentioned in hospital policies. (N1)*<br>• *More often, their mindset remains focused on how to solve problems after an adverse event has happened. As for this kind of forward-looking, preventive behavior, awareness is still quite weak. (N11)*
	Individual Barriers: Competence Limits and the Burden of Action	• *Even if channels are open, some are reluctant to offer more suggestions because they feel it might bring extra responsibility, or they're already too busy and don't want additional tasks. (N5)*<br>• *The root cause is often that nurses are unaware of the safety hazard... tracing it back, they likely did not realize it was a safety issue. (N5)*<br>• *...If they make a suggestion, it might mean they have to spend more time helping to implement it, so some nurses are reluctant to do so. (N8)*<br>• *There's also the workload pressure. Nurses are already very busy with their daily tasks, so taking on anything extra can feel overwhelming—they often simply avoid it. (N8)*<br>• *Nurses with a strong sense of responsibility tend to be more proactive and service-oriented. They usually pay more attention to unit safety and are more active in voicing their opinions and suggestions. (N7)*<br>• *Often, when nurses speak up, they may express themselves unclearly; what they intend to say and what is actually conveyed may differ. (N6)*<br>• *Some new nurses might miss potential hazards, or they might think an issue is minor and not realize the underlying safety risk. (N2)*

Themes	Sub-themes	Quotes
		• *Some people are outgoing and expressive, making it easier for them to share their thoughts; others are more introverted and less skilled in communication, so they speak up less. (N4)*<br>• *The nurse's personal understanding is also crucial. If training is inadequate, nurses might not realize how important speaking up for safety is. (N8)*<br>• *It relates to the nurse's personality. Some are naturally better at communicating with leadership, especially those adept at "managing upwards." If she feels a certain step poses a safety hazard, affecting her workflow or patient safety, she's willing to give feedback proactively, even offering constructive suggestions for improvement. (N10)*<br>• *Younger nurses or those with less solid professional knowledge might find it hard to make suggestions. They may lack confidence or be unsure what kind of questions are appropriate to raise. (N2)*<br>• *Then of course, there's the issue of work responsibility... a nurse's sense of duty is very important. Some nurses are meticulous and can spot potential safety hazards in their work; others might just mechanically follow procedures, paying little attention to patient safety—you know, thinking "I'll just complete my assigned tasks, do what I'm supposed to do," right? (N12)*<br>• *A major factor behind this is the time commitment. For nurses at work, beyond their routine duties, speaking up requires additional time and effort. For instance, if a nurse proposes a plan and the manager finds it good, they might assign you to implement it. During execution, you'd need to research, develop the plan, and then submit it for the manager's review. The manager might then say, "There's an issue with this part; revise it." So, you have to research again, make revisions, and resubmit. It's like invisibly piling a lot of extra work on yourself. (N6)*<br>• *It also relates to nurses' personal identification with or engagement in their work. Some nurses have the attitude of "the less trouble the better", possibly feeling that unit matters are not part of their personal career aspirations. (N2)*<br>• *Many nurses feel this doesn't really concern them, or they worry—if I raise this, management might adopt it, and that could mean extra work for me, right? Since suggesting things doesn't benefit them and might even create more work, they just stay quiet. (N10)*
	Environmental Inhibition: Hierarchical Pressure and Interpersonal Harmony	• *Some leaders might be more domineering or authoritarian, you know? In that kind of environment, nurses might feel too intimidated to speak up. (N10)*<br>• *If the relationship with the leader is poor, they definitely won't be proactive about making suggestions. That nurse might think, "I'm just here to do my job." With a strained relationship with the leader and feeling little investment in the unit's development, they might just go through the motions. When assigned a task, they might do it, but not with much care, believing their suggestions wouldn't matter anyway. (N6)*<br>• *They also have this psychological concern, thinking, "What if I raise this issue and it leads to punishment for someone? Or, after being punished, will they come back and think I'm the one who reported them?" That's a thought they might have. (N14)*<br>• *...From a nurse's perspective, there remains a sense of fear—they hesitate to approach leaders. Like when I was a staff nurse, I also avoided speaking with managers out of that fear, that intangible pressure. (N9)*<br>• *If the team atmosphere is poor, a nurse might raise a safety concern, only to end up feeling "I'm the only one responsible for fixing this." Then they'd be reluctant to speak up again. (N8)*<br>• *One must never be like some autocratic head nurses who have a "my word is law" attitude. If that's the case, staff definitely won't want to say anything... they just follow whatever you say and stop speaking up proactively. (N6)*<br>• *They might worry about being seen as "telling on" others—well, it's about the relationship between coworkers, and it*

Themes	Sub-themes	Quotes
		could make things really unpleasant. So that is a concern they have... (N14)<br>• *Then there's the relationship between doctors and nurses. If it's good, nurses pointing out issues with doctors' orders can be accepted more smoothly. But if tensions exist, nurses themselves have reservations and are less likely to dare to speak up. (N4)*<br>• *Besides the extra workload factor, I think it's also related to interpersonal relationships. Most nurses in our unit are women, who tend to be more sensitive. They might consider their social circles and relationships. If they make a suggestion, others might see it as them "stabbing someone in the back" or saying something bad behind someone's back. They worry it could affect their relationships with colleagues. (N6)*<br>• *If collegial relationships are fragile, even if a nurse identifies a problem, may dare not raise it directly out of fear that it might damage her relationship with colleagues. (N7)*
	Management Deficits: Institutional Gaps and Interdepartmental Barriers	• *Even if nurses have constructive suggestions, there are no effective channels to report them... (N7)*<br>• *Sometimes as a manager, I am reluctant to raise issues repeatedly. I feel like, 'Look, I've raised this so many times and it's still not resolved'; people will just start thinking you are being a nuisance. (N5)*<br>• *While there is some safety knowledge training, specific training on speaking up seems lacking. It's probably more focused on patient communication courses. (N4)*<br>• *Currently, although the hospital encourages reporting adverse events and has incentive mechanisms, dedicated initiatives specifically for speaking up for patient safety haven't really taken shape. (N1)*<br>• *I think the problem lies right here. Nurses' communication mainly occurs in daily work with patients and doctors, but a formal system to encourage nurses to suggest patient safety improvements to management hasn't been established. (N1)*<br>• *In the current hospital system, many issues and suggestions can't flow smoothly from the frontline to upper management. The capability and communication channels of the middle managers are crucial. (N5)*<br>• *Nowadays, it's often very difficult to implement suggestions because they don't get the necessary support. (N6)*<br>• *In outpatient settings, issues such as equipment problems or environmental hygiene… are recognised as real concerns. Yet when reported to the relevant departments with no active response or solution… it leaves everyone feeling that speaking up is futile—simply a waste of time. (N13)*<br>• *Some problems that seem urgent to nursing may not be a priority for other departments… For instance, I've recommended updating old equipment, but that doesn't necessarily lead to any action. (N5)*<br>• *Currently, we have no other special channels for collecting suggestions. From a hospital management perspective, there are no dedicated pathways or incentive mechanisms to encourage nurses to make recommendations. (N3)*<br>• *Our unit currently lacks specific incentive mechanisms... so nurses feel that if their suggestions aren't taken up, it's simply not worth speaking up. (N7)*<br>• *The hospital has no specific policy to encourage it…Beyond handling adverse events, after reporting, nurses are required to complete various forms and documentation... the process is quite complex. (N10)*<br>• *There don't seem to be any related policies at the hospital level. The hospital may value this type of work, but most of the responsibility is delegated to the departments... In short, the hospital's awareness in this area probably still needs to be strengthened. (N10)*

Themes	Sub-themes	Quotes
Theme 3: The Key to Empowerment: Building a Supportive Ecosystem	Scaffolding Bilateral Competence: Prioritizing Targeted Education	• *The hospital should provide guidance for head nurses and team leaders... specifically on how to effectively respond to suggestions... providing training on communication and management skills is necessary to improve efficiency and teamwork. (N10)*<br>• *If I, as a manager, lack sufficient understanding... I cannot guide nurses to express their views... If the manager does not value this concept, how can they mobilize the entire team...? (N11)*<br>• *If the head nurse themselves is unclear and can't teach the nurses what is right or wrong, then what meaningful safety voice can even exist in that unit? (N1)*<br>• *Regarding leadership, I believe head nurses need enhanced training. They must continuously learn, update their management philosophy and methods, and improve their management capabilities. (N1)*<br>• *Actually, I think first and foremost, nurses need awareness about safety voice—to understand what "safety" entails, what aspects it covers. Definitely, relevant training should be conducted, clearly explaining patient safety, healthcare worker safety, and so on. Safety voice isn't just about patient safety; it also concerns nurses' own safety. (N10)*<br>• *We need to develop training content. First, nurses need to know what this behavior called "safety voice" is. We need to help them understand that. Then, the training should also include real case studies to guide nurses. For example, using actual situations to illustrate: "Look, if I had intervened at this point, what might the patient's outcome have been?" Or, "If I hadn't detected this in time, leading to an unsafe event, what would the patient's prognosis have been?" Through these real cases, nurses can gain a deeper understanding of the impact of safety behaviors, enhancing their safety awareness and attitude. (N11)*<br>• *Everyone needs to learn, right? Learning is definitely important too—training, including on safety knowledge, and then emphasis. In our work, safety is the bottom line. It's about instilling that safety knowledge, right, the importance of safety. Safety overrides everything, right? Only when it's safe does all your work have meaning. If there are unsafe conditions, no matter how much you do, it's meaningless. (N12)*<br>• *Training for nurses is also very important, I think. Because you have to let them know the importance of this safety voice, what its significance is, right? And you also have to instill in them that this is not a form of "tattling" or "snitching." (N14)*<br>• *The hospital level certainly needs to make efforts too. First, it can strengthen training related to patient safety, including provincial or municipal organized safety education, whether online or offline. Because nurses' own understanding of safety is crucial. (N2)*<br>• *An ability-building mechanism needs to be established, making "safety voice" training a mandatory course. Leaders need to explain the importance of safety voice in staff meetings—why to speak up, what benefits it brings, and its impact on personal career development. This way, everyone will realize its importance, fostering an atmosphere of proactive voice. (N6)*<br>• *Training should not only improve theoretical and communication skills but also include value orientation. We need to tell nurses why safety voice is critical for patients and the unit. (N8)*<br>• *Currently, our hospital does not seem to have specialized training for safety voice... although there is case sharing for adverse events, it does not specifically revolve around how to raise safety concerns. (N8)*<br>• *First, we must let everyone understand the importance... through specialized training. We can use specific examples to illustrate where hazards can be found and then what measures to take. (N7)*

Themes	Sub-themes	Quotes
	Cultivating Psychological Safety: The Manager's Duty to Listen and Empower	• *Head nurses should appropriately delegate authority to nurses. For example, when a nurse identifies a problem and, after assessment by management, it's deemed solvable or the suggestion is feasible, they should be empowered to take initiative in managing and resolving it. This way, when they receive positive feedback, their sense of agency becomes stronger. (N13)*<br>• *Managers need to empower nurses... When nurses are granted the right to participate... they are no longer mere followers but key members... Empowerment helps them view issues from a holistic perspective... This sense of participation enhances confidence, motivating them to provide more valuable safety voice. (N8)*<br>• *The importance of speaking up can be emphasized repeatedly through leadership meetings, large or small, to encourage everyone to propose suggestions more actively in daily work. (N6)*<br>• *Leaders need to explain the importance of safety voice in staff meetings—why to speak up, what benefits it brings, and its impact on personal career development. This way, everyone will realize its importance, fostering an atmosphere of proactive voice. (N6)*<br>• *As managers, we also need to actively ask nurses if they have good ideas, listen to their opinions, and encourage them to express themselves. (N6)*<br>• *I think the most important thing depends on us, as nurse managers, to actively encourage and guide nurses to give us more feedback. If we can provide timely positive feedback, praise, encouragement, and even validate their opinions, then I believe clinical nurses won't just passively discover issues at work but will proactively use their own initiative to identify safety concerns. (N13)*<br>• *First, I think managers should use every opportunity to promote it. For instance, in morning meetings, weekly meetings, or monthly meetings, proactively ask nurses if they've noticed any patient safety issues or have any countermeasures. And if a nurse can offer constructive suggestions, they should be given recognition and praise. (N13)*<br>• *Head nurses should guide nurses to identify these problems, not just receive reported issues. They should enable nurses to proactively discover problems and propose improvements. (N5)*<br>• *…we need to create an environment where nurses dare to speak up and are encouraged to express their views… and we also need to guide their thinking. (N9)*<br>• *I think we need to be inclusive, to actively listen to nurses' thoughts and suggestions, and then use them to optimize processes and systems. At the same time, we should also provide resource support for nurses, like training and learning opportunities. Make them feel that their voices are heard. (N4)*<br>• *...the manager's own safety literacy has a huge impact... We must firmly hold the safety baseline... and use our actual actions to influence every nurse, making safety awareness a team habit. (N10)*<br>• *As a manager, you can't be impatient with everything they say… You have to listen patiently, because important issues might surface even from minor talk. (N5)*<br>• *(Sigh) This really isn't easy to handle. How to get nurses to be proactive (think)... From a leadership perspective, leaders must show approachability, can't be aloof and make people afraid to speak. (N1)*<br>• *Leaders can't be aloof. They definitely need to know what's happening clinically, what the conflicts and difficulties are. Then, they need to listen patiently and, from a leadership perspective, propose practical solutions, not shuffle responsibilities or make excuses. (N1)*

Themes	Sub-themes	Quotes
	Institutionalizing Responsiveness: Establishing Structural Guarantees	• *The institutional aspect is crucial... there should be a clear voice policy with distinct responsibilities... We must have a concrete, actionable plan. Every step should have a designated professional... rather than just being an empty title. (N7)*<br>• *To eliminate what they perceive as "snitching," I think we could establish a suggestion group or channel. Information could be collected electronically or anonymously. This anonymous setup would yield more genuine feedback and make people feel safer to speak up. (N14)*<br>• *If a suggestion is feasible and we can achieve the goal, we should formalize the resulting system and process. First, everyone needs to learn it, then implement it. After implementation, we need to evaluate to see how effective it is. Then we can pursue continuous improvement. (N4)*<br>• *If there were an information platform where everyone could see the status... that would make it much more open and transparent. (N4)*<br>• *Building information systems is also very important. (N6)*<br>• *Whether leaders respond to suggestions promptly is crucial. Timely feedback matters a lot—it's like in games, where the reward mechanism is key. Whether you give feedback immediately or only remember to do it much later can lead to very different outcomes. (N10)*<br>• *If nurses feel their suggestions receive no feedback or don't lead to actual change, they may become unwilling to voice opinions. This means the hospital should provide effective feedback channels for nurses and ensure every suggestion gets a response, thereby fostering a healthy safety culture. (N3)*<br>• *Most importantly, we need to track the implementation results of suggestions. If a suggestion isn't adopted, we should show full respect to avoid disregarding or dampening nurses' enthusiasm. Even if not adopted, we should clearly explain the reasons, maintaining respect for the nurse. (N2)*<br>• *Regular feedback is also important. After someone offers a suggestion, you can't ignore it and give no feedback. If someone raises an issue or suggestion and you don't respond at all, people will be reluctant to speak up in the future. So, regardless of whether the suggestion is adopted, a result should be fed back to them. (N6)*<br>• *First is verbal encouragement, then promoting good suggestions. Next are monetary incentives, mainly in the area of adverse events, but this is quite limited and based on hospital policy, not set by our nursing department. (N1)*<br>• *If the hospital actively encourages, and the department can also reward valuable suggestions, give them weight in performance evaluations, or even just offer verbal encouragement, it can all have positive effects. (N2)*<br>• *...consider incorporating constructive suggestions into unit performance evaluations... For nurses who frequently raise valid suggestions, we could give appropriate preference in year-end evaluations or external study opportunities. (N2)*<br>• *Leaders should make their stance clear, express support for safety voice, and set up incentive mechanisms within the department. This way, nurses will be more willing to speak up and offer their suggestions. (N7)*<br>• *The hospital's role is more about creating an atmosphere, fostering an environment, and introducing incentive policies. For example, if a department can reward nurses with time, the hospital level can also provide rewards in performance evaluations. Actually, not every minor issue needs a reward, but for some important, major practical problems, the hospital can offer support. (N9)*<br>• *I think if the hospital could provide more efficient communication channels, it would better promote voice... (N10)*<br>• *The hospital could set up dedicated channels, like a dean's suggestion box or anonymous feedback… This allows issues to be resolved harmoniously without straining relationships. (N10)*

Themes	Sub-themes	Quotes
		• *Or, if there are methods like anonymous suggestions, listening to nurses' voices through multiple channels can also help stimulate their voice behavior. (N2)*<br>• *Beyond the departmental level, we also need a higher-level channel. Sometimes, nurses' suggestions stop at the departmental level, which also limits their motivation. For instance, the hospital intranet, though some content isn't directly related to nursing safety behaviors, could provide a platform for nurses to voice opinions anonymously. Many nurses would likely be more willing to express their views. (N3)*<br>• *It would be even better if the hospital could open a dedicated "Patient Safety Mailbox." This mailbox could allow for anonymity options, and nurses could submit questions or suggestions through various methods without fear of blame, giving everyone an outlet for expression. (N2)*<br>• *Patient safety voice could also work this way, driven top-down, not necessarily waiting for nurses to proactively suggest from the bottom up. This top-down collection also gives nurses an opportunity. The key is still to give nurses a channel, but currently, there's no channel that connects top to bottom. (N3)*<br>• *Through different avenues, the channels must be open. If the channels aren't open, nurses naturally won't be willing to speak. (N5)*<br>• *Most importantly, the hospital should encourage them to do these things with an open and inclusive attitude, not by mandating that nurses must do it or by reacting negatively to individuals. (N9)*<br>• *Spiritual rewards. For example, having a "Safety Star" in the department (laughs), making them feel their work is meaningful. Also, at the department or hospital level, organizing competitions for "golden ideas" or sharing excellent patient safety voice cases. (N1)*<br>• *The first is providing rewards. Giving partial rewards in performance evaluations—for example, if you identify a hazard or offer a relatively constructive suggestion, I can reward you. I think this is acceptable too. (N14)*<br>• *First, there need to be several basic conditions: first, provide economic rewards, and the work environment should be comfortable; second, have emotional value, meaning recognition for nurses' feelings. After voicing a suggestion, if a nurse's idea is useful, it should be rewarded. If a nurse raises a problem and solves it, we should make them feel their contribution is recognized. (N5)*<br>• *I think the most important measure should be reflected in performance evaluations, especially incentive policies. If there's a reward mechanism, nurses will be more motivated to make suggestions. (N6)*<br>• *The hospital level could establish activities like a "Safety Month," commending departments or individuals who offer excellent suggestions, issuing certificates to increase nurses' sense of belonging and achievement. This can boost everyone's motivation and encourage more people to participate in safety voice. (N6)*<br>• *Incentive mechanisms also cannot be ignored. If a nurse's safety voice is adopted, the hospital can motivate them through praise, rewards, etc. We don't care much about the monetary amount of the reward; we value spiritual encouragement more. For example, praising contributing nurses at head nurse meetings—this kind of spiritual encouragement is often more meaningful than material rewards. (N8)*

Themes	Sub-themes	Quotes
	Normalizing the Atmosphere: Cultural Integration and Promotion	• *The hospital's cultural atmosphere is crucial—whether a safety culture is truly integrated into daily work, and whether related activities are in place to influence nurses' thinking and behavior, is also a very significant factor. (N11)*<br>• *Through skits, short plays, or similar dramatizations, we can show how problems are identified and risks are prevented. This helps everyone grasp the significance of safety voice more intuitively. (N8)*<br>• *The hospital needs to use culture as a guide, for instance, by launching awareness activities or a Speak Up for Safety Day to spread the idea of safety voice. (N8)*<br>• *Most importantly, the unit must emphasize building a safety culture atmosphere, making it clear that safety is the bottom line and cannot be ignored. (N11)*<br>• *Hospitals or units can share examples where nurses' safety voice prevented unsafe incidents, establishing role models for speaking up within the organization. (N13)*<br>• *First, leaders should start with the unit environment—ensuring safety layouts and signage are in place and visible, which helps create a safety-conscious atmosphere. Additionally, policies need updating; for example, safety management protocols should be revised, and regular training organized to enhance nurses' competencies. (N7)*<br>• *We can also promote it through static materials. For example, in unit corridors, we can set up safety bulletin boards to display suggestion cases or relevant safety knowledge. (N6)*<br>• *If someone gives a good safety suggestion that genuinely improves safety, we can share it via PPT in nurse managers' meetings for others to see. (N1)*<br>• *At the hospital level, I think we could organize activities to help build an atmosphere of speaking up for patient safety. (N1)*<br>• *Like the case-sharing sessions we already have, which usually discuss incidents that have occurred, especially valuable cases. But safety suggestions focus more on prevention—proposing improvements. We can draw on this format to encourage nurses to speak up, especially regarding how to prevent problems. (N7)*<br>• *I think we could set up a "Speak Up for Safety Day"—for example, selecting one day each month. Participation wouldn't be mandatory for every unit, but we could actively encourage it. On that day, we could discuss adverse events, daily safety precautions, and potential risks, encouraging everyone to share opinions and suggestions. If a nurse's safety suggestion is adopted, we could provide incentives or recognition, such as praise or public acknowledgment. (N8)*
